# Comparison of Single‐Breath and Multi‐Breath Xe‐MRI in the Longitudinal Assessment of Treatment in Children With Cystic Fibrosis

**DOI:** 10.1002/jmri.70242

**Published:** 2026-01-29

**Authors:** Faiyza S. Alam, Samal Munidasa, Brandon Zanette, Daniel Li, Sharon Braganza, Felix Ratjen, Giles Santyr

**Affiliations:** ^1^ Department of Medical Biophysics University of Toronto Toronto Ontario Canada; ^2^ Translational Medicine Program The Hospital for Sick Children Toronto Ontario Canada; ^3^ Division of Respirology The Hospital for Sick Children Toronto Ontario Canada

**Keywords:** cystic fibrosis, fractional ventilation, hyperpolarized 129Xe, lung clearance index, multiple‐breath washout

## Abstract

**Background:**

Elexacaftor/tezacaftor/ivacaftor (ETI) is a current standard therapy for pediatric cystic fibrosis (CF). Multiple‐breath washout ^129^Xe MRI (MBW Xe‐MRI) is improved following 1 month of treatment. However, the utility of MBW Xe‐MRI over extended ETI treatment and its comparison to single‐breath Xe‐MRI and pulmonary function tests (PFTs) in monitoring disease progression remains unclear.

**Purpose:**

To compare MBW Xe‐MRI and single‐breath Xe‐MRI in a small pediatric CF cohort at 1, 6, 12, and 24 months post‐ETI initiation.

**Study Type:**

Prospective longitudinal cohort study.

**Subjects:**

14 participants (7 female, median age 15.5 [14, 17] years) with CF undergoing ETI.

**Field Strength/Sequence:**

Xe‐MRI using a gradient echo sequence at 3T.

**Assessment:**

A total of 12 participants completed MBW Xe‐MRI, single‐breath Xe‐MRI, and PFTs (spirometry, N_2_ MBW) at ≥ 2 of 4 visits (1, 6, 12, and 24 months post‐ETI). Fractional ventilation (FV) and FV coefficient of variation (CoV_FV_) maps were calculated from MBW Xe‐MRI. Ventilation defect percent (VDP) was calculated from single‐breath Xe‐MRI.

**Statistical Tests:**

Longitudinal changes were analyzed using a linear mixed‐effects model (fixed effect: time, random intercept: participant). Significance via ANOVA F‐test, *p* < 0.05. Intra‐class correlation coefficients (ICC) were used to quantify between‐ and within‐subject variability.

**Results:**

Data completeness (total number of acquired data points divided by expected data points across 14 participants, 4 visits) was ≥ 75%. While PFTs/VDP remained stable over 24 months (ICC ≥ 0.93; linear mixed‐effects model of time effect for ppFEV_1_, LCI and VDP was not significant with *p* = 0.68, 0.13 and 0.12, respectively), CoV_FV_ demonstrated a small but significant increase (slope magnitude +0.001/month). Furthermore, two participants had elevated CoV_FV_ despite normal VDP. Finally, MBW Xe‐MRI metrics showed higher within‐subject variability than PFTs/VDP (ICC: FV = 0.41, CoV_FV_ = 0.55 vs. VDP/PFTs ≥ 0.92).

**Data Conclusion:**

CoV_FV_ may continue to evolve over 2 years in pediatric CF patients receiving ETI, particularly in individuals with persistent ventilation defects.

**Evidence Level:**

1.

**Stage of Technical Efficacy:**

2.

## Plain Language Summary

Clinical pulmonary function tests (PFTs) are insensitive to early cystic fibrosis (CF) lung disease in children, which limits their usefulness for evaluating new treatments such as elexacaftor/tezacaftor/ivacaftor (ETI). Multiple‐breath washout xenon MRI (MBW Xe‐MRI) creates images of lung function that may be more sensitive to early CF and has shown improvement after 1 month of ETI. This study examines how MBW Xe‐MRI performs over longer treatment (24 months) and how it compares with standard measures like PFTs. While other measures remained stable, MBW Xe‐MRI detected subtle disease progression over time and may help complement current tools for monitoring pediatric CF.

## 1 Introduction

Longitudinal clinical trials suggest that pulmonary function tests (PFTs), including spirometry and the nitrogen multiple‐breath washout test (N_2_ MBW) yielding the lung clearance index (LCI), remain stable for at least 24 months after elexacaftor/tezacaftor/ivacaftor (ETI) initiation in pediatric cystic fibrosis (CF) [1]. However, PFTs lack regional information, and spirometry, including the forced expiratory volume in 1 s percent predicted, ppFEV_1_, is often normal in younger children [2], limiting sensitivity.

Single‐breath hyperpolarized ^129^Xe MRI (Xe‐MRI) provides a regional measure of obstruction via the ventilation defect percent (VDP), which quantifies the proportion of poorly ventilated lung [3]. VDP is a widely utilized metric which has been shown to be repeatable [4, 5] and has demonstrated sensitivity to functional improvements as early as 1 month post‐ETI initiation [6, 7]. However, single‐breath Xe‐MRI only captures ventilation in a single static breath‐hold and is not directly reflective of dynamic ventilation abnormalities such as delayed gas clearance or regional trapping.

Multiple‐breath washout Xe‐MRI (MBW Xe‐MRI) can quantify fractional ventilation (FV) and its spatial heterogeneity (CoV_FV_), which may improve sensitivity to ETI treatment [8, 9, 10, 11, 12]. Both FV and CoV_FV_ derived from MBW Xe‐MRI have been shown to be feasible [8, 9, 10], repeatable [11], and improved following 1 month of ETI therapy in pediatric CF [12]. However, the utility of FV and CoV_FV_ over extended ETI treatment and how they compare directly to single‐breath Xe‐MRI and PFTs in monitoring disease progression remain unclear.

Thus, the aim of this study was to compare MBW Xe‐MRI and single‐breath Xe‐MRI in a small pediatric CF cohort at 6, 12, and 24 months post‐ETI initiation. A further aim was to assess and compare longitudinal trajectories and variability of FV, CoV_FV_, VDP, ppFEV_1_, and LCI.

## 2 Materials and Methods

This study extends the observation of a cohort studied 1 month after ETI initiation [12] to 6, 12, and 24 months post‐initiation. Ethical approval for this study was obtained from the SickKids Research Ethics Board (REB#10000063021, Clinical‐Trials.gov NCT04391322). Participants from the original pilot study (which itself was an extension of a larger, multi‐site study called HyperPOlarized Imaging for New Treatments (HyPOINT), REB#1000077493, Clinical‐Trials.gov NCT04259970) were re‐consented for longitudinal monitoring up to 24 months. Briefly, the original cohort were 9–18 years old at baseline (< 1 week before initiation of ETI), with a confirmed CF diagnosis and medical prescription to begin ETI therapy.

At each follow‐up visit, participants performed spirometry (yielding ppFEV_1_) and N_2_ MBW (yielding LCI) according to American Thoracic Society (ATS) and European Respiratory Society (ERS) standardized protocols [13, 14]. Participants also underwent both single‐breath Xe‐MRI and MBW Xe‐MRI using gradient‐recalled echo (GRE) sequences as previously described [4, 11, 12]. Briefly, single‐breath Xe‐MRI images were acquired after a 6–8 s initial breath‐hold of hyperpolarized xenon gas using 10–14 coronal slices (4 × 4 × 15 mm^3^), followed by proton MRI to obtain thoracic cavity estimation. For MBW Xe‐MRI, three sequential images (~7 s) were acquired for flip angle and T_1_ correction followed by tidal breathing of room air with interleaved breath‐holds (~5 s apart) at tidal inspirations during which single non‐selective coronal slices of the lungs were acquired (~1 s, 7.5 × 7.5 mm^2^ in‐plane) until signal was depleted, ~5–8 images. Detailed methods are described elsewhere [12].

Image processing for single‐breath Xe‐MRI and MBW Xe‐MRI was semi‐automatic and performed by FA (5 years experience in Xe‐MRI) and SM (7 years experience in Xe‐MRI) using MATLAB ver. R2024a (MathWorks, Natick, MA, USA) as described by Alam et al. [11, 12]. Briefly, for MBW Xe‐MRI, fractional ventilation (FV) was calculated from registered washout images on a per‐pixel basis from a mono‐exponential curve fit to the washout signal decay, representing gas clearance per breath ranging from 0 to 1.0; 0 representing no gas turnover after one breath, and 1.0 representing complete gas turnover after 1 breath. The CoV_FV_ was calculated by taking the standard deviation divided by the mean in a 3 × 3 image kernel for every FV voxel, generating a map representing regional heterogeneity of FV across the lungs. For single‐breath Xe‐MRI, VDP was calculated from co‐registered xenon and proton MR thoracic cavity images using a semiautomated segmentation algorithm [4], with defect regions determined as those being less than 60% of the mean signal [3].

### 2.1 Statistical Analysis

To assess longitudinal trajectories, linear mixed‐effects models were used with time as a fixed effect and participant as a random intercept to account for repeated measurements. The fixed‐effect slope represents the rate of change in each metric per month. Statistical significance of the time effect was assessed using an F‐test from the model ANOVA, with *p* < 0.05 indicating significance. The intra‐class correlation coefficient (ICC) was used for each metric to quantify between‐subject versus within‐subject variability across all visits. ICC values were categorized as excellent (> 0.90), good (0.75–0.90), moderate (0.50–0.75), or poor (< 0.50). Normal (i.e., healthy reference) values were obtained from literature as follows: ppFEV_1_ > 80%, LCI < 7.3 [15], VDP: 2.65 [2.32 3.58] [4], FV: 0.35 [0.31 0.50] [11], and CoV_FV_: 0.067 [0.057, 0.077] [11].

## 3 Results

### 3.1 Participant Enrollment and Data Completeness

**FIGURE 1:**
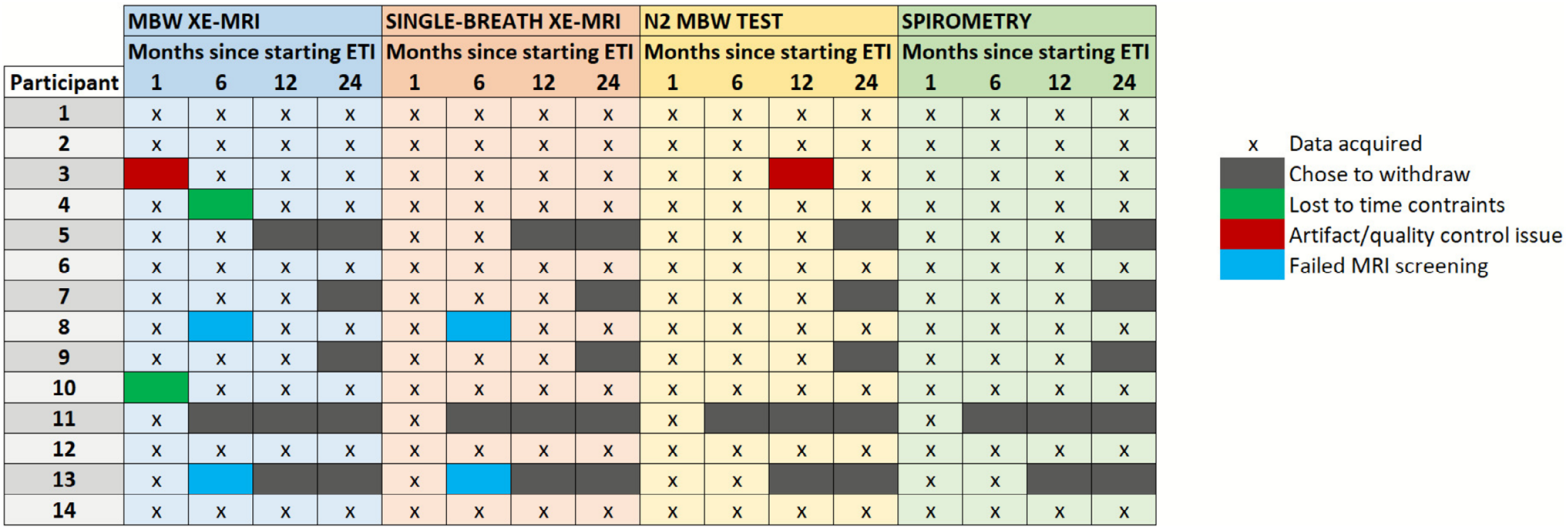
Overview of data availability for multiple‐breath‐washout xenon MRI (MBW Xe‐MRI), single‐breath Xe‐MRI, nitrogen multiple‐breath washout (N_2_ MBW) test, and spirometry across visits at 1, 6, 12, and 24 months following elexacaftor/tezacaftor/ivacaftor (ETI) initiation. Each row represents a participant, with colored cells indicating reasons for missing data (gray, withdrawal; green, lost to time constraints; red, artifact/quality control issue; blue, failed MRI screening) and “*x*” indicating successful data acquisition.

A total of 14 participants with median age 15.5 [interquartile range (IQR): 14, 17] years were reconsented. Out of these 14 participants, 12 had at least 2 out of 4 total visits (at 1, 6, 12, and 24 months post‐initiation of ETI) and were included in the analysis. Individual demographic information for all 14 participants is included in Table 1. Five participants completed all 4 MBW Xe‐MRI, 8 completed all 4 single‐breath Xe‐MRI and LCI, and 9 completed all 4 ppFEV_1_ (Figure 1). Overall, data completion rate (total number of acquired data points divided by total number of expected data points across 14 participants and 4 visits) for MBW Xe‐MRI was 75%, and for VDP, LCI, and ppFEV_1_, completion rates were 80%, 84%, and 86%, respectively. At 1‐month post‐ETI, participants had normal ppFEV_1_ (median [IQR] 91 [83104.5]), normal LCI (6.7 [6.4–8.9]), slightly elevated VDP (5.6 [3.6–9.8]), normal FV (0.42 [0.41–0.46]) and normal CoV_FV_ (0.060 [0.051–0.073]).

**TABLE 1:** Demographics, as well as metrics at 1 month after beginning elexacaftor/tezacaftor/ivacaftor (ETI) therapy for all 14 participants, ordered by descending baseline FEV_1_%.

Demographics															
Participant	1	2	3	4	5	6	7	8	9	10	11	12	13	14	Average
Age	16	18	15	13	12	14	15	17	18	16	16	15	17	14	15.5 [14 17]
Sex	F	F	M	M	F	M	F	M	M	M	F	M	F	F	7 M / 7F
BMI	22.6	23	34.2	19.6	24.2	17.9	18.1	19.5	23.2	21.5	21.9	19	21.6	21.6	21.6 [19.5 23]
Race	Black/African	Asian	White	White	White	White	White	White	White	Other	White	White	Asian	White	10 white/4 non‐white
Prior Modulator	No	No	No	No	Yes	Yes	Yes	No	No	No	Yes	Yes	No	Yes	6 yes / 8 no
Genotype	F508del/G542X	F508del/heterozygous	DeltaF508/c.3873 + 2 T>C	F508del/F508del	F508del/G551D	F508del/F508del	F508del/F508del	F508del/G542X	F508del/heterozygous	DeltaF508/c.1882G>A	F508del/F508del	F508del/F508del	C.16531655delCTT/c.1837 T>G	F508del/F508del	6 homozygous/8 heterozygous
Pancreatic Sufficiency	No	No	Yes	No	No	No	No	No	Yes	No	No	No	No	No	2 yes / 12 no

Metrics 1 month after beginning ETI															
FEV_1_ (%)	116	114	111	107	102	99	94	88	88	87	85	81	78	73	91 [85.5106]
FEV_1_/FVC (%)	104	93	92	106	105	97	97	95	87	75	95	80	85	78	94 [85.5 97]
LCI	6.41	8.84	7.40	6.70	6.67	8.91	6.25	6.43	11.4	8.21	7.42	5.94	9.46	6.51	7.0 [6.4 8.7]
VDP (%)	7.6	11.9	4.0	3.7	3.5	6.2	5.8	5.5	14.3	16.8	4.9	3.3	17.8	3.1	5.6 [3.8 10.8]
FV	0.37	0.47	—	0.41	0.51	0.41	0.43	0.41	0.46	—	0.45	0.47	0.29	0.42	0.43 [0.41 0.46]
CoV_FV_	0.039	0.060	—	0.045	0.059	0.071	0.047	0.075	0.087	—	0.056	0.063	0.089	0.058	0.060 [0.051 0.073]

*Note*: Values are reported as median [IQR] where relevant.

Abbreviations: BMI = body mass index; CoV_FV_ = coefficient of variation of FV; FEV_1_ = forced expiratory volume in 1 sec; FV = fractional ventilation; FVC = forced vital capacity; LCI = lung clearance index; VDP = ventilation defect percent.

### 3.2 Longitudinal Trajectories

**FIGURE 2:**
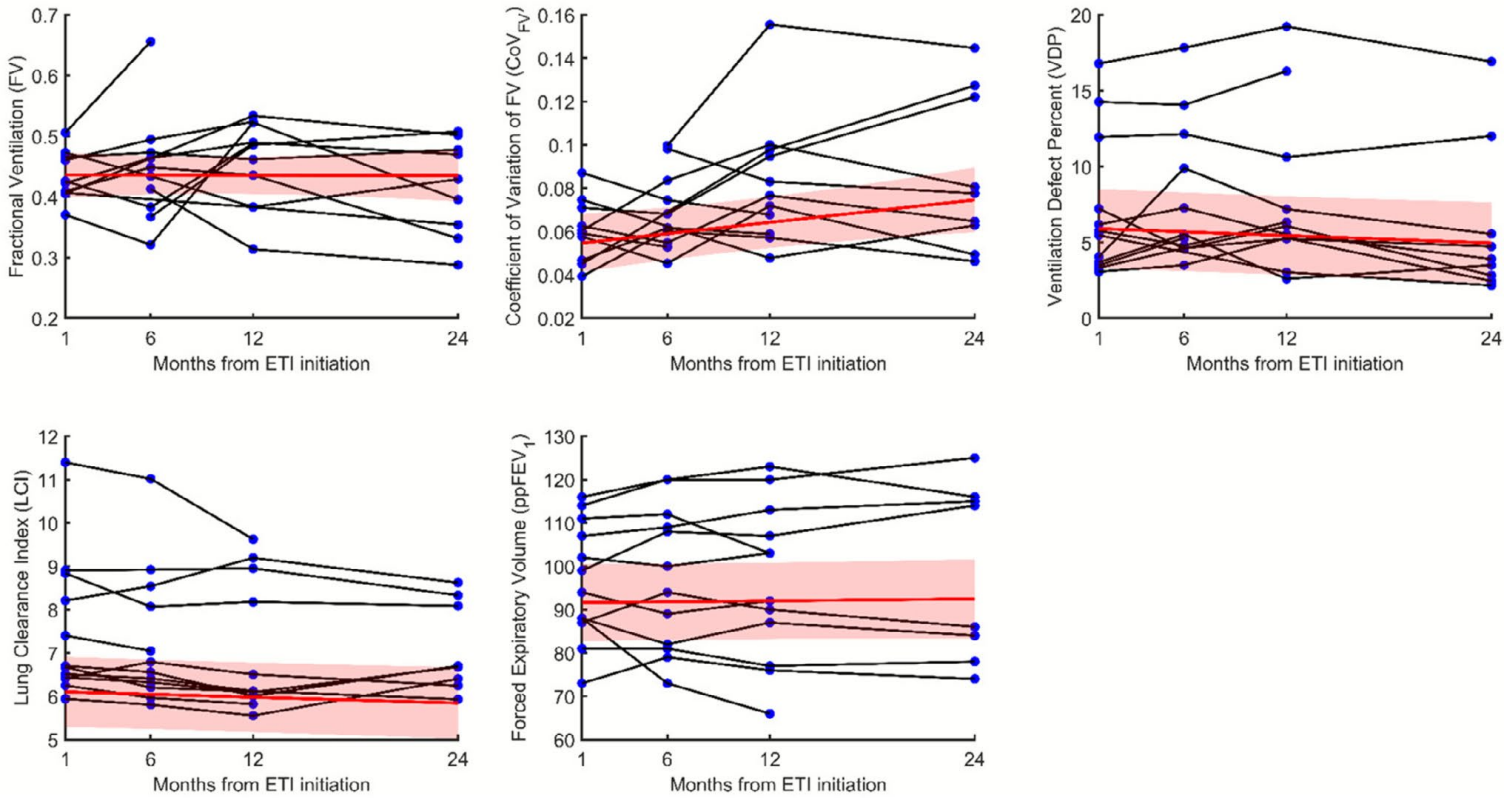
Individual subject line plots for (A) fractional ventilation (FV), (B) coefficient of variation of FV (CoV_FV_), (C) ventilation defect percent (VDP), (D) lung clearance index (LCI) and (E) forced expiratory volume in one second, percent predicted (ppFEV_1_) from 1, 6, 12, and 24 months post‐initiation of ETI therapy. Red lines represent the line of best fit, modeled from a linear mixed model with subject as a random effect and time as a fixed effect. The shaded red area represents the 95% confidence interval of the line of best fit.

Figure 2 shows longitudinal trajectories for all metrics beyond the initial 1‐month timepoint. Only CoV_FV_ had a significantly increasing slope over time, with a slope magnitude of +0.001/month. No significant trajectories were observed for FV (*p* = 0.98), VDP (*p* = 0.12), LCI (*p* = 0.13), or ppFEV_1_ (*p* = 0.68).

### 3.3 Longitudinal Variability

MBW Xe‐MRI metrics showed greater variability compared to PFTs and VDP. FV had the lowest ICC at 0.41 [0.25 0.88], reflecting the highest within‐subject variability relative to the cohort. ICC for CoV_FV_ was moderate at 0.55 [0.40 0.90]. In contrast, PFTs and VDP had excellent ICCs: VDP = 0.92 [0.90 0.97], LCI = 0.94 [0.92 0.99], and ppFEV_1_ = 0.93 [0.90 0.98].

### 3.4 Comparison Between Single‐Breath and MBW Xe‐MRI

**FIGURE 3:**
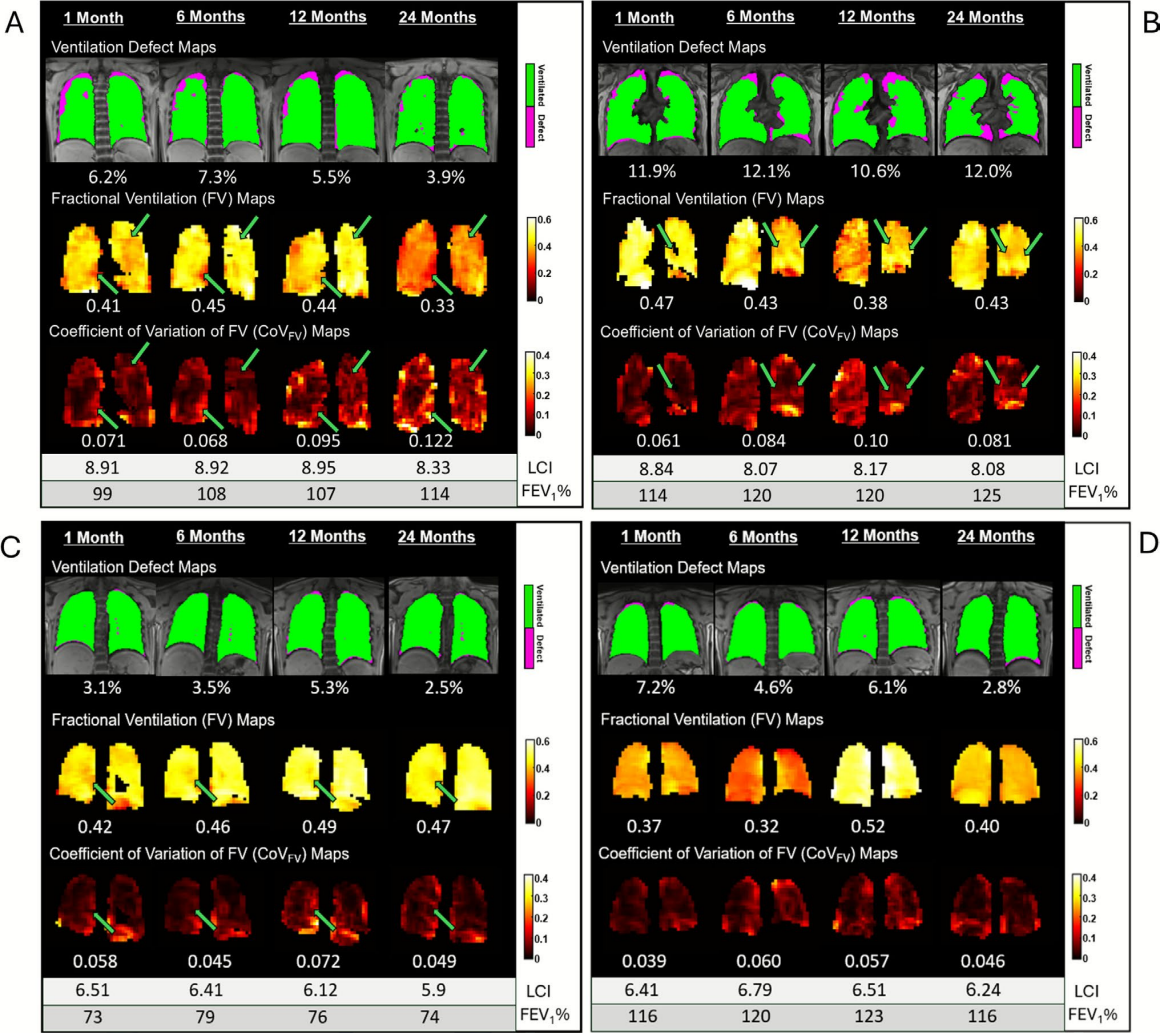
Ventilation defect, fractional ventilation (FV) and coefficient of variation (CoV_FV_) maps of four representative CF participants in all four visits. Lung clearance index (LCI) and forced expiratory volume in one second, percent predicted (FEV_1_%), values for each participant are shown beneath their respective images. Two participants with a trajectory towards increasing CoV_FV_ over time (A: 14‐year‐old male; B: 18‐year‐old female) and 2 participants without the trend (C: 14‐year‐old female; D: 16‐year‐old female). Green arrows point to an example of a persistent feature between visits.

Figure 3 displays representative examples of ventilation defect, FV, and CoV_FV_ maps in four individual participants illustrating the range of ventilation patterns observed. Figure 3A,B shows examples of participants with ventilation defects that did not resolve by 1 month post‐ETI and persisted up to 24 months. Figure 3C,D show examples of participants with no visible ventilation defect at any visit between 1 and 24 months post‐ETI. Notably, those with visible persistent ventilation defects were observed to have increasing CoV_FV_ over time, while this same trend was not observed in those without visible defects at 1 month. Despite widely heterogeneous global FV within individuals, repeatable features were observable in FV/CoV_FV_ in some participants (Figure 3, green arrows).

**FIGURE 4:**
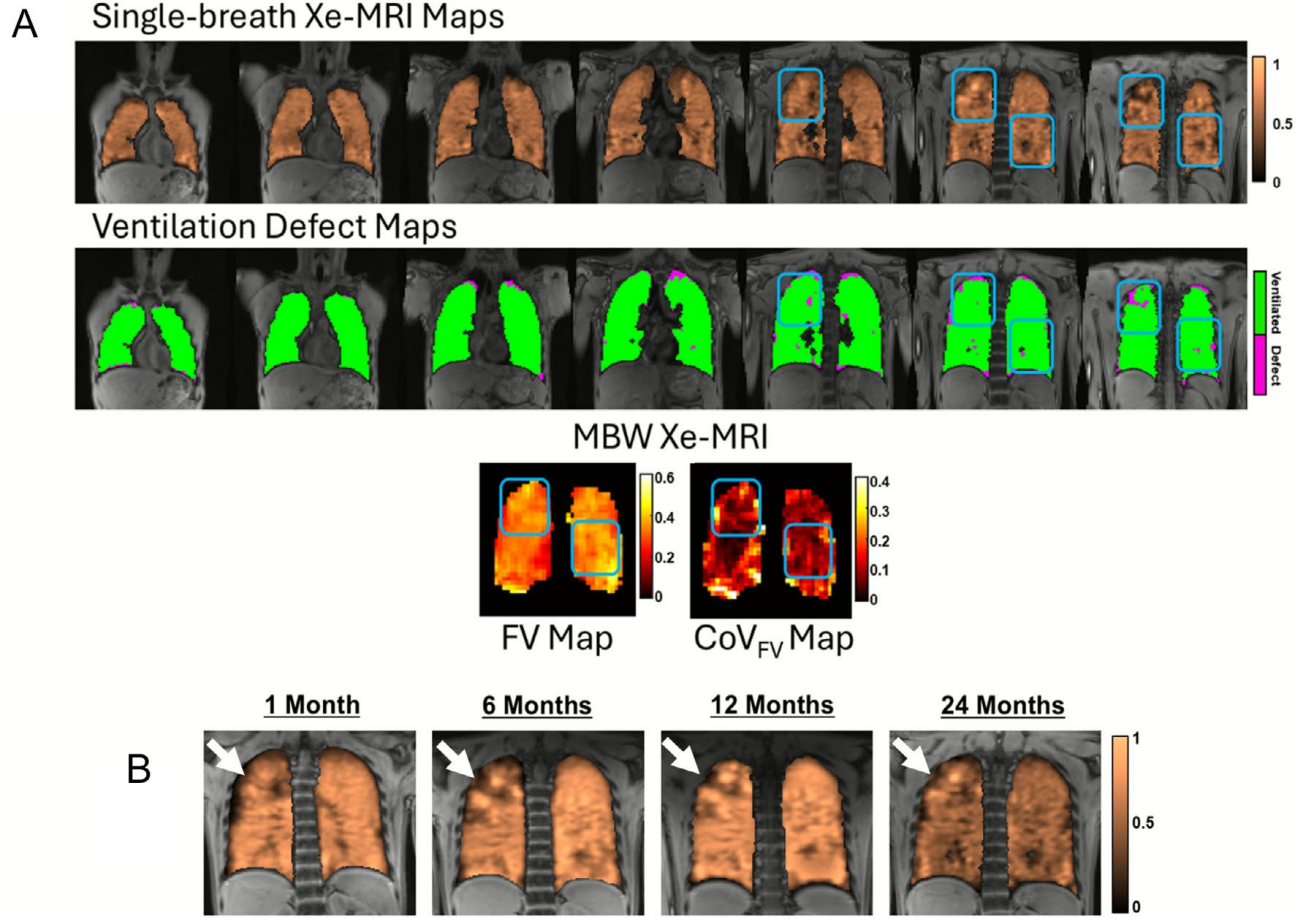
A: Single‐breath Xe‐MRI‐derived ventilation and ventilation defect maps, as well as MBW Xe‐MRI‐derived fractional ventilation (FV) and coefficient of variation of FV (CoV_FV_) maps for the 24‐month visit of the 14‐year‐old male participant in Figure 3A. Blue regions highlight visible heterogeneity in the ventilation maps that do not appear in the defect maps, but appear in FV and CoV_FV_ maps. B: Single‐breath Xe‐MRI‐derived ventilation maps for the same participant across all visits. The area of compensatory ventilation (white arrows) developed after 1 month and remained persistent up to 24 months.

At each visit, VDP and CoV_FV_ were generally concordant (i.e., when VDP was elevated above normal, CoV_FV_ was also elevated, and vice versa) except in two individuals, including the participant in Figure 3A, where CoV_FV_ was markedly elevated while VDP was within normal limits. Both individuals had visible, persistent ventilation defects in one or two slices but were otherwise ventilated, resulting in low multi‐slice VDP. To explore this observed divergence between VDP and CoV_FV_, Figure 4 shows a few slices of the single‐breath ventilation map alongside a few slices of the ventilation defect map for the representative participant in Figure 3A at 24 months, compared to corresponding FV/CoV_FV_ maps. Blue circles mark regions of ventilation heterogeneity, specifically regions of elevated signal intensity close to regions of low signal intensity, that are captured by FV and CoV_FV_ maps and corroborated by single‐breath ventilation maps but not quantified in the ventilation defect map. Figure 4B shows the signal intensity images for this participant across all slices, demonstrating increasing signal intensity around defect regions over time. LCI remained slightly elevated in this participant across all visits.

**FIGURE 5:**
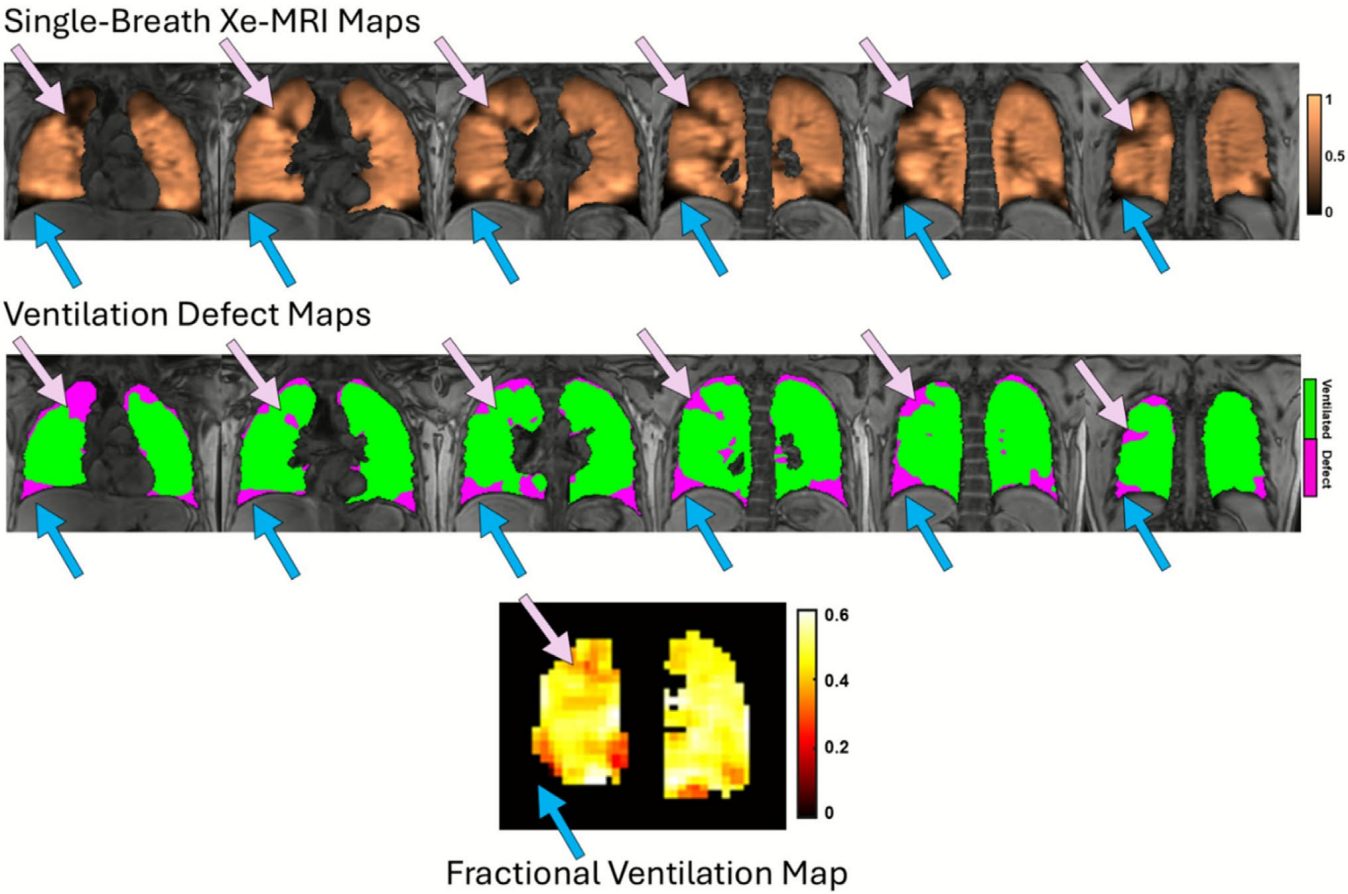
Single‐breath Xe‐MRI‐derived ventilation and ventilation defect maps, as well as MBW Xe‐MRI‐derived fractional ventilation (FV) map at 1 month in an 18‐year‐old male participant. Blue arrows point to an example of a ventilation defect in the ventilation defect map that is present across all slices and thus has no corresponding FV calculation. Purple arrows point to an example of a ventilation defect in the ventilation defect map that is partially‐obstructed across the anterior/posterior projection and is represented as a region of low FV in the FV map.

Differences in acquisition influenced the characterization of defects. MBW Xe‐MRI was acquired via single‐slice projection along the A/P direction, while single‐breath Xe‐MRI used multi‐slice imaging. As shown in Figure 5, regions that were absent in FV maps (i.e., not computed due to lack of signal) were concordant with ventilation defects spanning the full slice thickness, whereas regions with low FV were concordant with partial defects along the A/P direction.

## 4 Discussion

This study provided a direct longitudinal comparison of single‐breath and MBW Xe‐MRI in the context of an extended ETI treatment paradigm in pediatric CF over 2 years. While group‐level trajectories for FV, VDP, LCI, and ppFEV_1_ remained stable over 24 months, CoV_FV_ showed a small but significant increase over time. Measures of spatial ventilation heterogeneity, such as CoV_FV_, may be more sensitive to evolving changes in regional ventilation not detected by PFTs and VDP. Moreover, while CoV_FV_ and VDP were generally concordant at each visit, two individuals with elevated CoV_FV_ despite normal VDP demonstrate the potential of MBW Xe‐MRI to detect regional abnormalities that may not be well captured by VDP. However, small sample size, image acquisition differences, and high within‐subject variability of MBW Xe‐MRI suggest these findings should be considered exploratory. Further studies in larger cohorts are needed to validate the complementary role of MBW Xe‐MRI for long‐term monitoring in pediatric CF.

PFTs exhibited excellent reliability with no significant trajectory after 1 month, suggesting measurement stability. This is consistent with previous clinical trials, which observed that PFTs remained stable up to 2 years post‐initiation of ETI [1, 16]. Similarly, VDP exhibited excellent reliability with no significant trajectory, indicating that ventilation defects also remained stable post‐ETI.

In contrast, MBW Xe‐MRI had low measurement stability with poor/moderate ICCs for FV and CoV_FV_, despite repeatable features visible across visits. For global FV, trajectories were widely heterogeneous across visits within individuals. The lack of a significant trajectory in FV over time may be in line with results of previous studies, which found that FV failed to reliably differentiate between healthy subjects and CF [9, 10, 11] with non‐selective MBW Xe‐MRI. Additionally, while FV has previously been shown to be repeatable over 1 month in stable CF [11], this may have been due to the shorter time frame and inclusion of participants with more measurable disease (i.e., pre‐ETI). Growth (i.e., aging) related changes in tidal volume/effort may have further contributed to longitudinal variability, as FV is known to be highly effort dependent [11]. Future studies should explore the impact of age‐ and growth‐related changes on MBW Xe‐MRI. CoV_FV_ captures the spatial heterogeneity of FV rather than absolute FV and thus may retain stability even if absolute FV is more variable. The low ICCs for both FV and CoV_FV_ highlight the need for further technique optimization. Tracking and/or controlling inhaled gas volumes, which was not done here, has been shown to improve FV repeatability, quantitative accuracy, and correlations with PFTs [17]. MBW Xe‐MRI would also benefit from multi‐slice/volumetric acquisitions, which may increase sensitivity to regional changes in the A/P direction [18].

Interestingly, increasing CoV_FV_ was only observed in individuals with persistent defect regions on the ventilation defect map, suggesting a potential link between the two. This study showed heterogeneity in signal intensity images, particularly around defects, with elevated signal intensity possibly reflecting a compensatory increase in ventilation towards healthier lung regions in response to local ventilation loss. This has been described in patients undergoing lung volume reduction surgery (LVRS) as remaining lung tissue adapts to maintain overall ventilation [19]. This redistribution may progressively increase over time. Such compensatory changes may not be captured by N_2_ MBW, as LCI is primarily sensitive to slow‐emptying regions at the tail of the washout curve. If surrounding regions compensate by becoming faster‐emptying while the number of slow‐emptying regions remains unchanged, LCI may remain stable [20].

The varying signal intensities in single‐breath Xe‐MRI may reflect a static snapshot of the ventilation process and can be considered a proxy for ventilation dynamics. However, VDP analysis is often limited to raw signal intensities, typically interpreted through threshold‐based segmentation. While VDP derived using 60% thresholding has been shown to be best at differentiating between CF and healthy controls [21], it reduces the complexity of ventilation to a binary defect classification that masks changes within regions labeled as ventilated. Other approaches such as linear binning may better quantify changes in ventilation signal intensity distributions with thresholding multiple bins to explore ventilation defect, hypo‐ and hyper‐intensities relative to a healthy population distribution [22]. Nevertheless, the underlying signal remains influenced by non‐physiological factors such as inhaled volume, polarization level, and coil sensitivity, making it difficult to ascribe direct physiological meaning to individual voxel intensities. MBW approaches over several breaths attempt to correct for this. Although outcomes (e.g., FV) in this study are reported with summary statistics (e.g., means), the underlying voxel values represent outputs of a physiological model (i.e., gas washout), rather than strictly signal magnitude.

While correlations between metrics may be of interest, longitudinal correlation analyses of changes across the 24‐month period are complicated by the minimal change in PFTs over this time period. As a result, correlations of their changes over time would primarily capture measurement noise rather than meaningful biological relationships. Furthermore, the variable missing data across visits and participants results in different subsets of patients at each timepoint, making correlation analyses statistically problematic and potentially misleading. Notably, correlations between MBW Xe‐MRI metrics and PFTs have been established in prior work. In the previous study of this cohort, changes in CoV_FV_ from baseline to 1‐month post‐ETI correlated significantly with changes in both VDP (*R* = 0.93, *p* < 0.001) and LCI (*R* = 0.92, *p* < 0.0001), but not ppFEV_1_ [12]. In a separate repeatability study, absolute values of CoV_FV_ correlated with VDP (*R* = 0.45, *p* < 0.01) and LCI (*R* = 0.56, *p* < 0.001) at single timepoints, but not ppFEV_1_ [11]. FV did not correlate with any metric in either study.

## 5 Limitations

In this study, single‐breath images were acquired using multiple slices, while MBW maps used a single A/P projection to preserve signal across breaths. This limits the sensitivity of FV and COV_FV_ maps, as well as the direct comparison of defect regions to regions of abnormal FV. Multi‐slice MBW Xe‐MRI in this population is feasible (as recently demonstrated [18, 23, 24]) and should be further investigated to enable slice‐to‐slice comparison with single‐breath Xe‐MRI.

Another limitation of the approach in this study is that single‐breath and MBW Xe‐MRI both employ the same gas administration wherein a single bolus of xenon gas is inhaled prior to imaging, which does not fully equilibrate throughout the lungs. As a result, the comparatively small volume of inhaled xenon predominantly reaches the same well‐ventilated regions in both techniques. Thus, MBW Xe‐MRI is more heavily weighted to fast‐filling/emptying regions. Furthermore, it does not probe slow‐emptying regions in the context of > 10 washout breaths known to be relevant to CF lung disease [25] due to fast irrecoverable loss of the hyperpolarized signal. As a result, FV derived from MBW Xe‐MRI reflects an earlier portion of washout than LCI, and the two measures are not directly comparable. A thermally polarized ^19^F gas such as perfluoropropane (PFP), when mixed with oxygen, enables extended (~20 breaths) washout (and washin). This enables detection of slow‐filling regions which may improve cohort differentiation and tracking of disease progression [26, 27, 28, 29]. Recent preliminary work comparing MBW Xe‐MRI to MBW PFP‐MRI suggests that some defects apparent using xenon may actually correspond to slow‐emptying regions rather than complete obstructions when measured with PFP [29].

A further limitation of FV/CoV_FV_ in this study is that these metrics did not account for obstructed/non‐ventilated regions due to lack of signal. As a result, individuals with substantial ventilation defects may appear to have normal FV and low/homogeneous CoV_FV_. Combining both defect burden and ventilation dynamics (e.g., by registering MBW Xe‐MRI maps to ^1^H thoracic images) may provide a more comprehensive assessment of ventilation than single‐breath or MBW Xe‐MRI alone.

Since MBW Xe‐MRI relies on coached breath‐holds instead of exact delivered volumes, image registration is necessary to align washout images. While coaching typically achieves adequate reproducibility [11], substantial inter‐breath tidal volume variations can introduce registration errors that may contribute to the observed variability in FV and CoV_FV_. Horn et al. mitigated this by incorporating an MRI‐compatible pneumotachograph to exclude data when a tidal volume change of > ±15% occurred [30]. The absence of a pneumotachograph in the current study represents a technical limitation.

Finally, this study is exploratory and limited by the small sample size of 12 heterogeneous participants. MBW Xe‐MRI has been shown to be repeatable over 1 month [11], and the current study provides initial data on variability over longer intervals (up to 24 months). However, MBW Xe‐MRI had the lowest completion rate compared to PFTs and VDP. As the secondary outcome measure (next to VDP), it was usually the first to be truncated in the event of time considerations. As such, the extent to which the observed variability is predominantly driven by pathophysiological change, measurement variability, or sample size limitations remains unclear.

## 6 Conclusion

This study showed that in pediatric CF patients undergoing ETI therapy over 2 years, PFTs and VDP remained stable while CoV_FV_ demonstrated a small but significant increase. These findings suggest that regional ventilation heterogeneity may continue to evolve, particularly in individuals with persistent ventilation defects.

## Funding

Supported by The Canadian Institutes of Health Research (CIHR) operating and project grants (MOP 123431, PJT 153099) and The Cystic Fibrosis Foundation (CFF). Faiyza Alam is supported by a Restracomp fellowship from the Hospital for Sick Children.
